# Phosphorus and Nutrition in Chronic Kidney Disease

**DOI:** 10.1155/2012/597605

**Published:** 2012-05-30

**Authors:** Emilio González-Parra, Carolina Gracia-Iguacel, Jesús Egido, Alberto Ortiz

**Affiliations:** Division of Nephrology and Hipertensión, IIS-Fundación Jiménez Díaz, Autonoma University, 28040 Madrid, Spain

## Abstract

Patients with renal impairment progressively lose the ability to excrete phosphorus. Decreased glomerular filtration of phosphorus is initially compensated by decreased tubular reabsorption, regulated by PTH and FGF23, maintaining normal serum phosphorus concentrations. There is a close relationship between protein and phosphorus intake. In chronic renal disease, a low dietary protein content slows the progression of kidney disease, especially in patients with proteinuria and decreases the supply of phosphorus, which has been directly related with progression of kidney disease and with patient survival. However, not all animal proteins and vegetables have the same proportion of phosphorus in their composition. Adequate labeling of food requires showing the phosphorus-to-protein ratio. The diet in patients with advanced-stage CKD has been controversial, because a diet with too low protein content can favor malnutrition and increase morbidity and mortality. Phosphorus binders lower serum phosphorus and also FGF23 levels, without decreasing diet protein content. But the interaction between intestinal dysbacteriosis in dialysis patients, phosphate binder efficacy, and patient tolerance to the binder could reduce their efficiency.

## 1. Introduction

Daily phosphorus ingestion is approximately 1200 mg, of which 950 mg are absorbed. Around 29% of body phosphorus is located in bone, and less than 1% is in the blood, which is the phosphorus that is quantified in clinical practice. Most phosphorus (70%) is located intracellularly and is interchangeable. Phosphorus is removed by two systems, the gastrointestinal tract, (150 mg/day) and the urine (800 mg/day) [1]. Ingestion of phosphorus by an individual with normal renal function results in immediate phosphaturia probably mediated by phosphatonins of intestinal origin [2]. A positive phosphorus balance recruits other phosphatonins. The first one, faster and transient, is parathyroid hormone (PTH) and the second one, slower and lasting, is Fibroblast Growth Factor 23 (FGF23).

Patients with renal impairment progressively lose the ability to excrete phosphorus. Decreased glomerular filtration of phosphorus is initially compensated by decreased tubular reabsorption regulated by PTH and FGF 23. This compensation leads to a normal urinary excretion of phosphorus in 24 h and in maintenance of normal serum phosphorus [3]. However, the adequacy of 24 h urinary phosphorus excretion is difficult to interpret, since we do not know the phosphorus ingested, and, as renal function deteriorates, a positive phosphorus balance results.

FGF23 is a 251 amino acid phosphatonin, which promotes phosphaturia by decreasing phosphorus reabsorption through inhibition of Na/P cotransporter type II activity in proximal tubules and by decreasing phosphorus absorption in the gut by inhibiting generation of active vitamin D in proximal tubules through inhibition of renal 1 alpha hydroxylase. Reduced active vitamin D facilitates PTH secretion, which further promotes renal phosphorus excretion [4–6]. FGF23 is released by bone generating the concept of an osteo-renal axis for phosphorus balance control that has changed traditional paradigms [4].

## 2. Protein Intake and Phosphorus

There is a close relationship between protein and phosphorus intake [7]. Proteins are rich in phosphorus so most of the scientific societies recommend reducing protein intake from early stages in patients with chronic renal failure, to reduce the input of phosphorus. One gram of protein has 13–15 mg of phosphorus of which 30–70% is absorbed through the intestine. Thus, an intake of 90 g of proteins a day results in absorption of 600–700 mg of phosphorus daily. In hemodialysis the net positive phosphorus balance in 48 hours is 1200–1400 mg/day, of which dialysis only removes 500–600 mg/session. Thus, there are two good reasons to restrict protein intake in chronic renal disease. On one side, a low dietary protein content slows the progression of kidney disease, especially in patients with proteinuria [8]. In addition,a protein-restricted diet decreases the supply of phosphorus, which has been directly related with progression of kidney disease and with patient survival. A restricted protein diet has additional advantages (Table 1). In advanced chronic kidney disease (CKD) most guidelines recommend a diet containing 0.6 to 0.8 g protein/kg/day based on meta-analysis demonstrating its efficacy [9]. This restriction is safe nutritionally and metabolically [10].

After initiating dialysis the dietary protein intake should be increased. Hemodialysis patients with higher protein intake have improved survival, despite higher phosphorus intake [11]. In a post hoc analysis of the HEMO study patients without dietary protein restriction have a better survival than those eating a protein-restricted diet [12]. However, a high protein intake is associated with a high intake of phosphorus and the latter is associated with increased cardiovascular mortality. This relationship holds even when adjusted for serum phosphorus, type and dose of phosphorus binders, and protein and energy intake [13]. For this reason an adequate protein intake should be associated with a restriction of dietary phosphorus.

The Food and Nutrition Board of the Institute of Medicine recommends a diet with 700 mg/day of phosphorus in healthy people, and 1250 mg/day in children and pregnant women [14]. However, a lower intake is recommended in the renal patient to reduce. The same recommendations advise restricting food additives containing phosphorus.

## 3. Phosphorus Absorption and Protein of Different Origins

Phosphorus in foods is found in different forms. Organic phosphorus associates with proteins has a low absorption. By contrast absorption of inorganic phosphorus found in additives and preservatives is very high, above 90%. A large amount of phosphate are added to foods as preservatives as well as from common beverages such cola, with a high phosphate content [15]. However, organic phosphorus from plant protein has a lower absorption than phosphorus from animal protein, ranging from 40 to 50%. The reason is that phosphorus from plants is in the form of phytates and mammals lack phytases. Phosphorus in animal protein is in the form of organic phosphate, which is readily hydrolyzed and absorbed [16].

 In rats with slowly progressive renal failure fed a casein-based or a grain-based protein diet, both of which with equivalent total phosphorus contents had the same serum phosphorous levels. However, the casein-fed animals had increased urinary phosphorus excretion and elevated serum FGF23 compared to the grain-fed rats [17].

In a crossover trial 11 patients with CKD stages 3-4 ingested a diet with animal or vegetable protein for 7 days. Animal protein intake increased serum phosphorus and FGF23 more than vegetable protein intake [18]. The simple recommendation is to reduce preservatives and additives in the first place, favor foods rich in vegetables, reduce meat, and avoid convenience foods.

However, not all animal proteins and vegetables have the same proportion of phosphorus in their composition. There are tables and graphics depicting the amount of phosphorus contained in various foods [19]. Adequate labeling of food requires showing the ratio of phosphorus (in mg) to protein (in grams). The ratio ranges from <10 to >65 mg/g. Cheese and soft drinks have a high ratio. This ratio is recommended by KDOQI guidelines and has several advantages [19].

- It is independent of the portion of food served.
- It simultaneously represents the contribution of phosphorus, and protein.
- It draws attention to the phosphorus-rich foods, especially soft drinks and additives and are not proteins.

However, this ratio does not provide information on the bioavailability of phosphorus from different sources. Patients with CKD should be prescribed a low phosphorus, low inorganic phosphorus and low phosphorus/protein ratio diet, and with a proper protein content to improve the attractiveness of food.

The Mediterranean diet, until recently widespread in Spain, has a low phosphorous content and has been shown to reduce plasma homocysteine, serum phosphorus, microalbuminuria, and cardiovascular risk [20]. Food additives and preservatives are rich in phosphorus [21]. Additives account for about 1000 mg/day of phosphorus on average in the American diet. This amount is important in patients on hemodialysis [22]. Cheese and soft drinks have a high content of phosphoric acid, in addition to a high phosphorus-to-protein ratio [23].

Phosphorus intake is now a hallmark of poor quality food. Individuals with lower socioeconomic status and a lower income have higher serum phosphorus possibly due to the abuse of preprepared meals and fast food containing additives [24].

## 4. Low-Protein Diet and Malnutrition

The diet in patients with advanced-stage CKD has been controversial throughout the history of Nephrology. CKD is associated with protein calorie malnutrition [25]. A diet with a too low-protein content can favor malnutrition and increase morbidity and mortality [7]. However a low-protein diet can slow the progression of renal disease. While normoproteic or high-protein diet may increase uremic symptoms and hyperphosphatemia. A delicate balance should be sought. A low-protein diet in CKD has the following potential advantages (Table 1): decreases uremic symptoms [11], improves phosphorus control [11], delays initiation of dialysis [9], does not increase the risk of protein malnutrition if accompanied by essential amino acid supplement [26], does not increase mortality in patients with low-protein diet after starting dialysis [27], and protects against oxidative stress which may aggravate progression of CKD [28].

In dialysis, protein intake should not be restricted despite a higher intake of phosphorus, since the risk of protein malnutrition and mortality exceeds that of hyperphosphatemia [11]. When dialysis patients are prescribed a low-protein intake, actual protein intake is frequently lower than expected, possibly because of the difficulty in implementing the diet. Thus, a recommended intake of 0.3–0.6 g/kg/day protein is estimated to result in an actual intake of 0.48–0.84 g/kg/day [26, 29]. Implementation of a low-protein diet requires a dedicated staff, with nurses, dietitians, and a close monitoring by nephrologists. However, in hemodialysis patients net phosphorus balance on a normoproteic diet is positive even after deducting the phosphorus removed during the dialysis session. Hemodialysis removes 800 mg phosphorus/session (2400 mg/week). Thus, a protein intake of 1 g/kg BW/day as recommended will result in an estimated weekly net balance of phosphorus of 2000 mg.

Savica el al. suggest to the patients undergoing periodic HD that they must ingest a quantity of protein of 1,2–1,4 gr per Kg of body weight in the day and in other hand that they must ingest a quantity of phosphate no more than 800 mg per day. So 1,2–1,4 gr of protein correspond to 1.450 mg–1600 mg of phosphate and 800 mg of phosphate correspond to 0.6 mg per Kg b.w. per day. If the patients follow the suggestion to ingest no more 800 mg of phosphate in the day they are at high risk for malnutrition. In fact both dialysis treatment and phosphate binders are unable to remove the phosphate ingested [15]. We report that 74% of CKD pts ingest beverages and if we considere this evidence we can calculate a weekly net positive balance of phosphate of about 2.800 mg. This weekly quantity of phosphate is very dangerous for calcification in CKD pts [30].

The association between low-protein intake and increased mortality in dialysis patients suggests that alternative methods are needed to reduce phosphorus absorption, since high phosphorus is associated with mortality. There are two main alternatives. One is the use of specific nutritional supplements high in energy and protein content, but low in phosphorus. This diet allows maintaining an adequate nutritional status, without altering the serum phosphorus, and without need for higher phosphorus binders [31]. The second alternative is nutritional education of the patient. This includes greater attention to additives and preservatives, to the contribution of phosphorus from different protein foods, so that the diet is based on low phosphorus/protein ratio ingredients, as well as the proper and early use of phosphorus binders [11].

## 5. Phosphorus Binders

In a major retrospective study patients treated with phosphorus binders before entering dialysis and phosphate above 3.7 mg/dL, had a better long-term survival than those in whom binders were initiated after initiation of dialysis. Similar results were obtained when binder use in the first 90 days of dialysis was compared with later initiation of binders [32]. The authors speculated that the observation might be explained by modulation of direct effect of phosphorus or compensatory mechanisms such as FGF23 on patient survival [33]. However, this reduction in mortality was not observed in incident dialysis patients treated with calcium-containing binders, either calcium acetate or calcium carbonate [34]. Phosphorus binders lower serum phosphorus and also lower FGF23 levels. Indeed in early CKD binders may result in reduced FGF23 levels in the absence of changes in serum phosphorus [35].

The purpose of therapy with usual phosphate binders is either to limit the absorption of dietary phosphorous intake and to maintain phosphatemia in normal range. Mostly of them act by binding phosphate in gut and eliminating it in the stool. Authors have observed that the salivary phosphorous ratio in hemodialysis patients is more than doubled compared with healthy controls [36, 37]. Same authors have demonstrated that salivary phosphate binders, like chitosan-loaded chewing gum, reduced serum phosphate [38].

## 6. Different Efficacy and Tolerance between Captors: Binding to Bile Salts

There are different phosphorus binders for clinical use, which have different binding power and side effects. In addition, the binding power and side effects may differ between individuals and impact on efficacy [39]. The main side effects relate to digestive tolerance in around 15–20% of patients. [40, 41]. In case of intolerance, modification of the prescribed binders may reduce the side effects and decrease the absorption of phosphorus. Lanthanum carbonate has been successfully used in controlling hyperphosphatemia in patients with intolerance to other binders [42].

To better understand the causes of reduced efficiency and digestive intolerance, we must understand the peculiarities of the digestive tract in uremic patients. CKD patients frequently have intestinal dysbacteriosis, which may be multifactorial [43] (Table 2).

 Disbacteriosis promotes the release of products of bacterial metabolism that may enter the blood and originate uremic toxins such as phenols, indoles, and amines. Uremic toxins may contribute to an increased risk of cardiovascular and bone disease.

 Uremic patients may have a poor digestion and malabsorption of protein, carbohydrates, and fats [44]. Potential causes are bacterial overgrowth or disorders of the exocrine pancreas or biliary function. Indeed, uremia is associated with high plasma levels of peptides such as secretin, pancreatic secretagogues and gastrin, and an abnormal composition of pancreatic secretion, including low bicarbonate and amylase levels.

 Some phosphate binders bind to bile salts. Ten out of 49 dialysis patients studied had bacterial overgrowth as assessed by the lactulose test, and this was associated with dyspepsia [44]. Sevelamer exacerbated dyspepsia, but supplementation of oral pancreatic enzymes improved symptoms and the phosphate binder effectiveness. This highlights the interaction between intestinal dysbacteriosis, phosphate binder efficacy and patient tolerance to the binder.

 Phosphorus binders binding to bile salts may interfere with soluble molecules that require biliary salts for absorption. In this sense sevelamer binding to bile salts results in reduced cholesterol absorption and lower serum LDL-cholesterol and in reduced vitamin D absorption [45].

## 7. Conclusions

Phosphate overload and hyperphosphatemia have emerged as risk factors for vascular calcification, cardiovascular mortality, left ventricular hypertrophym and progression of chronic kidney disease. Normoprotein or high-protein diet may increase uremic symptoms and hyperphosphatemia, but low protein intake and increased mortality in dialysis patients suggests that alternative methods are needed to reduce phosphorus absorption. An adequate nutritional status includes greater attention to additives and preservatives, to the contribution of phosphorus from different protein foods, a diet based on low phosphorus/protein ratio ingredients as well as the proper and early use of phosphorus binders.

## Figures and Tables

**Table 1:** Consequences of dietary protein restriction in advanced chronic kidney disease.

Reduces proteinuria.	
Improves lipid control	
Reduces uremic toxins and acids	
Reduces oxidative stress	
Improves insulin resistance	
Reduces phosphorus load	

**Table 2:** Causes of CKD patients intestinal dysbacteriosis.

Dialysis patients eat less fiber than healthy individuals, in part because dietary restrictions that includes the reduction of fruit and vegetables to avoid an overload of potassium.	
Uremia results in intestinal acidification.	
Certain drugs, such as antibiotics and phosphate binders alter the intestinal flora.	
Bowel dysfunction may cause constipation or increased intestinal transit time.	
The metabolism and absorption of proteins is altered and this may lead to malnutrition.	
